# Audiologic Outcomes with Auditory Brainstem Implantation Including Successful Open Set Speech Perception with Bilateral Implantation

**DOI:** 10.3390/audiolres16040095

**Published:** 2026-06-23

**Authors:** Douglas M. Bennion, Alicia Williams, Claire Perrin, Joshua Lee, Peter Eckard, Philipp Verpukhovskiy, Madeline Gibson, Rick A. Friedman, Marc S. Schwartz

**Affiliations:** 1 Department of Otolaryngology, Head and Neck Surgery, University of California San Diego, San Diego, CA 92093, USA; douglas-bennion@uiowa.edu (D.M.B.); aaw002@health.ucsd.edu (A.W.); clperrin@health.ucsd.edu (C.P.); rafriedman@health.ucsd.edu (R.A.F.); 2 Department of Otolaryngology, Head and Neck Surgery, University of Iowa, Iowa City, IA 52242, USA; 3 Department of Neurological Surgery, University of California San Diego, San Diego, CA 92093, USA

**Keywords:** auditory brainstem implant, neurofibromatosis, vestibular schwannoma, auditory outcomes

## Abstract

**Background/Objectives**: For patients with profound deafness resulting from auditory nerve pathology, as in Neurofibromatosis type 2, auditory brainstem implantation (ABI) can restore meaningful acoustic input. The literature reporting real-world results for ABI users is limited, especially regarding patients with bilateral implants. Here, we provide an updated report on the audiologic outcomes among all ABI patients treated at a tertiary institution, including high-performing bilateral ABI users. **Methods**: In this updated and expanded retrospective case series, audiologic outcomes were reviewed in sixteen consecutive patients who underwent ABI placement by a single neurosurgeon-neurotologist team at our center since 2018. Implantation in four of these patients was on their second side after having undergone first side implantation prior to receiving care at our hospital. Main outcome measures were sound awareness (sound-field threshold testing) and speech understanding (pattern perception, spondee, open-set speech testing). **Results**: Sound awareness was achieved in 100% of patients (16/16) using an average of 12 electrodes (range 7–20). Persistent non-auditory sensations were reported by 12.5% (2/16). Postoperative speech differentiation (with or without lip-reading) was experienced in 87.5% (14/16). Two second-sided ABI recipients experienced exceptional outcomes as high-performing outliers: one achieved 57% audio only and 86% audio + visual hearing in noise test (HINT) sentence scores; the second bilateral user scored 92% with auditory-only input. **Conclusions**: ABI represents a viable option for patients who are at risk of developing bilateral profound deafness resulting from auditory nerve disruption. Second sided device implantation is safe and has the potential to significantly improve auditory outcomes.

## 1. Introduction

For patients with conditions disrupting bilateral cochlear nerves such as Neurofibromatosis type 2 (NF2), traditional options for aural rehabilitation provide limited or no benefit at advanced disease stages. William House and William Hitselberger developed the first auditory brainstem implant (ABI) at the House Ear Institute and Clinic in the 1970s [1], with the first implant being placed in a patient in 1979 [2]. In comparison to outcomes possible with cochlear implantation, which began nearly two decades earlier, expectations for auditory performance with ABIs have been moderated. The ABI system has since evolved from a single-channel to a multi-channel system, from which most patients report environmental sound awareness and better lipreading capabilities [3]. However, ABI outcomes remain inferior to traditional cochlear implants, with most users relying on lip-reading, speech recognition technology, or sign language as their main forms of communication. Recent studies have shown that patients can achieve varying levels of pattern and phoneme discrimination with modern ABIs, in addition to environmental sound awareness, though only a small subset are able to achieve open-set speech discrimination [4,5].

The Food and Drug Administration (FDA) approved the use of the multichannel ABI in 2000 for individuals 12 years and older with a diagnosis of NF2. Off-label implantation for non-NF2 patients followed in the United States and in Europe, including in adults and children with bilateral temporal bone fractures, bilateral complete cochlear ossification, or bilateral cochlear nerve aplasia [6,7,8,9]. Other clinical trials have explored indications in pediatric patients including complete labyrinthine aplasia, cochlear nerve aplasia, cochlear aplasia, cochlear hypoplasia, cochlear nerve hypoplasia, cochlear malformations, ossified cochlea, auditory neuropathy, and trauma [10,11,12].

Here, we review the ABI outcomes at our single tertiary care center where NF2 patients who undergo microsurgical tumor resection are offered concurrent placement of an ABI. A previous report from our center by de Cos et al. [13] provided an initial review of NF2 patients who underwent ABI implantation with results indicating that 9 out of the 11 patients demonstrated benefits specifically in improvement of aided warble-tone audiometry. However, long-term and audiometric outcome data in ABI implantation patients remains largely unexplored. In this case series, we investigated an expanded cohort of 16 patients with more extended follow-up and audiometric outcomes, including several patients who underwent second-sided ABI placement to address this gap in knowledge.

## 2. Materials and Methods

### 2.1. Study Population

Included patients had a diagnosis of NF2 and underwent a translabyrinthine craniotomy for removal of cerebellopontine angle tumors and concurrent ABI placement, with one exception for a patient with profound bilateral sensorineural hearing loss after traumatic brain injury complicated by meningitis. This patient did not receive benefit from prior unilateral cochlear implantation and a retrosigmoid approach was used for ABI placement. All patients underwent ABI implantation by the same neurosurgeon, and all ABI implantations performed at our tertiary-care center from 2018 to 2024 were included in this retrospective review.

### 2.2. Study Design, Ethical Approval, and Statistical Analyses

**Figure 1:**
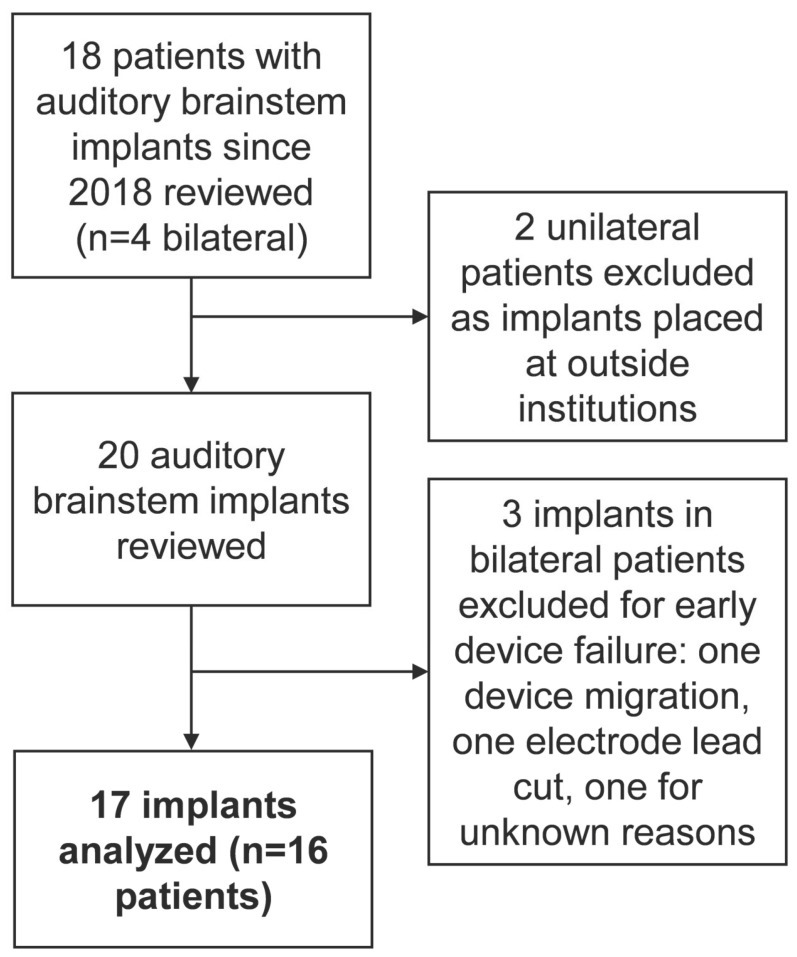
Patient inclusion and exclusion flowchart.

For this retrospective case series, medical records of all patients with ABI devices who received care at our institution from 2018 to 2024 were reviewed, including clinical and audiological records. This resulted in the identification of 16 patients with 17 functional ABIs (see Figure 1). Descriptive statistics of patient data included measurement of central tendency, variability, and frequencies. ABI-aided warble tone audiometric sound field thresholds are presented as average ± standard error of the mean (SEM). Simple logistic regression analysis was performed using Graphpad Prism (GraphPad Software Inc., San Diego, CA, USA) to examine the relationship between the number of active electrodes versus (1) current user status, (2) achievement of any amount of open-set speech recognition on consonant-nucleus-consonant (CNC), and (3) HINT sentence scores.

### 2.3. Description of Surgical Approach to ABI

Our surgical approach aligns with methods detailed in previously published reports [11,14]. For all cases, non-invasive nerve monitoring is routinely utilized, including monitoring of the cochlear nerve using electrically evoked auditory brainstem response (eABR) recordings. A standard translabyrinthine approach is performed, including developing a subperiosteal pocket for placement of the device receiver–stimulator. After tumor removal, compression of the brainstem may obscure the anatomy in some cases, heightening the importance of microdissection techniques with careful attention to landmarks. These include looking inferiorly from the facial and vestibulocochlear nerve root entry zones to identify the root entry zone of the glossopharyngeal nerve. The flocculus of the cerebellum may need to be gently displaced to bring into view the lateral aperture of the 4th ventricle, with a tuft of choroid plexus just dorsal to the glossopharyngeal nerve emanating through the foramen of Luschka in some cases. After dorsal displacement of the choroid plexus, the tenia choroidea, a soft tissue arachnoid band, may need to be delicately divided to allow access to the lateral recess of the 4th ventricle through the foramen of Luschka. The ependymal surface of the 4th ventricle is readily identifiable just deep to the level of the tenia.

Electrode array insertion into the lateral recess of the 4th ventricle is then performed. Using micro-cottonoids for gentle dissection and retraction especially of the choroid, the anterior and superior walls of the lateral recess can be identified. The opening of the lateral recess is usually just wide enough to place (not slide) the array into the lateral recess and advanced until the lateralmost electrode contacts can be seen to be in direct contact with the anterosuperior surface of the lateral recess at the point at which the brain surface curves ventrally. This places the mid portion of the array over the expected region of the cochlear nucleus complex. It is important to avoid excessive medial placement of the array, especially in cases where expected decompression of the brainstem may result in more medial shifting of the array over time. The array is then bolstered in place using pieces of teased Teflon pledget placed posteriorly and packing the space of the foramen of Luschka. The essential step of eABR testing is then performed, with adjustments made to final array positioning based on the number and region of electrode contacts that are found to be activated during testing. Following confirmation of array placement, closure commences with dural re-approximation, abdominal fat for obliteration of the mastoid cavity, and placement of a titanium reconstruction plate that is modified to avoid any contact or impingement on the electrode as it passes from deep within the mastoidectomy cavity to the subperiosteal pocket.

### 2.4. Description of Audiologic Approach to ABI Mapping and Assessment of Outcomes

All patients implanted at our center in this study received ABI 541 devices from Cochlear Ltd. (Sydney, Australia) Given that many patients travel from far distances for surgical care at our center, follow-up for device programming and audiologic outcome measurements were obtained at variable intervals. Device activation was performed as early as 6 weeks postoperatively, over two days. Initial stimulation and testing of each electrode in the array was performed in the postoperative anesthesia care unit with concurrent cardiac telemetry to obviate risk of aberrant device interference with cardiorespiratory brainstem centers. Day 2 of activation, as well as subsequent post-activation programming visits, was in the clinic. Subsequent audiological evaluations were performed as part of programming visits at various intervals from 1 to 2 months up to several years that were dictated by (1) patient availability to travel, and (2) performance. These included ABI-aided warble tone audiometry, early speech perception (ESP), and auditory spondee identification. ESP testing employs a hierarchical approach that assesses speech differentiation beginning with pattern perception, then spondee identification, and finally monosyllable identification. Positive ESP was defined as having at least pattern recognition. Auditory abilities with the auditory brainstem implant (ABI) were assessed using both open-set and closed-set word lists. Closed-set speech perception measures included early speech perception, and auditory spondee identification. Open-set measures included AzBio Sentences, the Hearing in Noise Test (HINT), and Consonant-Nucleus-Consonant (CNC) word testing. Warble-tone behavioral audiometry was utilized to determine sound awareness across various frequencies. Speech perception testing was conducted in a hierarchical manner, progressing from fundamental auditory perception to more complex speech recognition tasks (AzBio). To assess the benefit of speechreading, speech tests were administered in auditory-only, auditory-visual, and visual-only conditions. The selection of speech tests was guided by the patient’s auditory performance. If pattern perception was demonstrated, spondee recognition was assessed next, followed by more complex tasks as appropriate. If patients exhibited difficulty with simpler auditory tasks, they were provided time for aural rehabilitation following protocols for cochlear implant recipients. A follow-up assessment was conducted to reassess their progress.

Aided testing was performed with the patient wearing the ABI processor on the side being tested. When contralateral hearing was normal or near-normal, masking was presented to the contralateral ear via insert during testing to rule out contribution from the better-hearing ear to results obtained. Speech stimuli were presented using monitored live voice. For the auditory-only test condition, the tester’s face was obstructed to prevent the patient from lipreading. Auditory + visual condition allowed the patient to hear the speech stimuli and see the tester speaking the sentences. Visual-only condition consisted of seeing the tester without any audio stimuli.

## 3. Results

We identified 16 patients who underwent ABI at our institution since 2018 (see Figure 1 and Table 1). Of these 16 patients, one underwent implantation for an indication other than NF2: traumatic brain injury complicated by meningitis. As expected in NF2 patients, tumors were large, with an average largest dimension of 2.9 cm.

Among the 16 implants placed at our institution during the study period, the initial position of the electrode array was adjusted intraoperatively in four (25%) based on feedback from intraoperative eABR recordings. The average number of active electrodes after programming was 12. There were no ABI-specific postoperative complications, though facial nerve paresis was experienced by four patients (two HB grade 6, one HB grade 4, and one HB grade 3), and one patient suffered a cerebrospinal fluid leak requiring lumbar drain followed by ventriculoperitoneal shunt placement. Two patients experienced persistent non-auditory sensations with device use: one with dizziness after wearing the processor for long periods of time, and a second who experienced uncomfortable left arm sensations.

**Figure 2:**
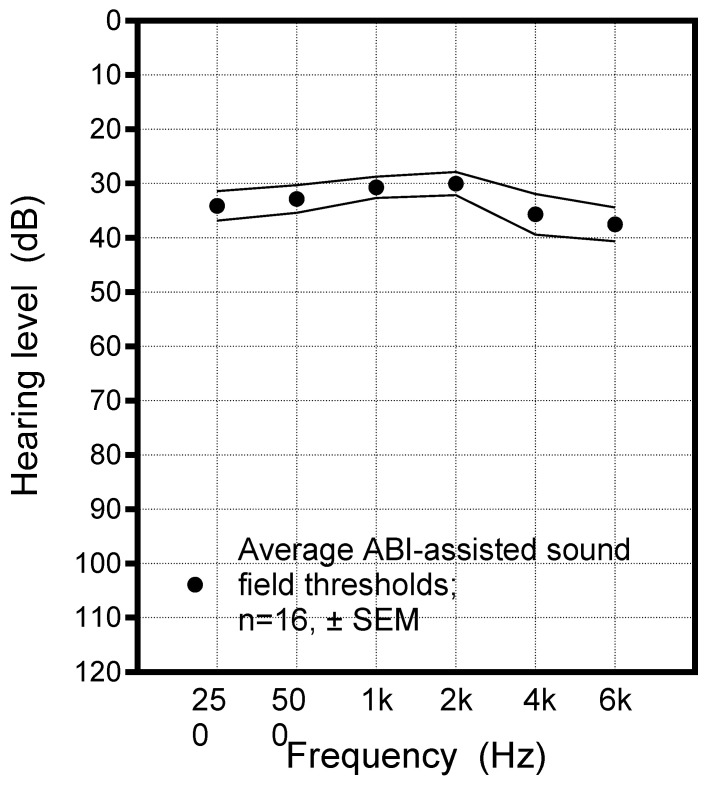
Average ABI-aided sound field thresholds of fifteen patients (16 ABIs). Lines are ± standard error of the mean.

Postoperative audiologic outcomes were generally favorable (Table 2). All patients (including subsequent non-users) reported subjective sound awareness, and 87.5% (14 of 16) patients reported improved lip reading with device use and 87.5% (14 of 16) reported at least early speech understanding after implantation. ABI-aided sound field thresholds, assessed in 15 patients, revealed average sound awareness consistent with mild hearing loss across all frequencies (Figure 2). Speech perception testing was performed in 14 patients using different test materials based on each patient’s performance: speech pattern perception, spondee word recognition, CNC words and phonomes, or HINT sentences (Table 3). The widely variable level of speech perception performance among our cohort is reflected by the different numbers of patients able to engage with the different test materials, which ranged from basic (pattern perception) to complex tasks (hearing in noise testing, HINT). As expected, auditory + visual scores exceeded auditory-only scores in all cases. Logistic regression analysis showed a higher number of active electrodes was associated with open-set speech recognition using CNC or HINT lists, which was achieved at some level in eleven implants, though this did not reach significance (*p* = 0.09).

At the last follow-up, ten out of sixteen patients reported ongoing daily use of their ABI. Importantly, among the six non-users, all retained intact contralateral hearing, so they were not dependent on their ABI for sound awareness. Logistic regression analysis of the number of active electrodes and current user status did not reveal a significant association (*p* = 0.48).

Among our cohort of sixteen ABI patients, four underwent second-sided implantation. Three of these four cases involved patients who discontinued use of their first ABI. One patient was unable to use the first ABI due to device failure from electrode array migration. Another patient had initial ABI placement at another center prior to his second-sided implant at our center and had stopped using the initial ABI due to mostly non-auditory sensations and intact contralateral hearing at that time. A third patient experienced electrode lead damage during subsequent ventriculostomy drain placement before first-side device activation. Following second-sided implantation, the patient achieved high auditory function, with HINT scores of 57% in the auditory-only condition and 86% in the auditory + visual condition.

**Figure 3:**
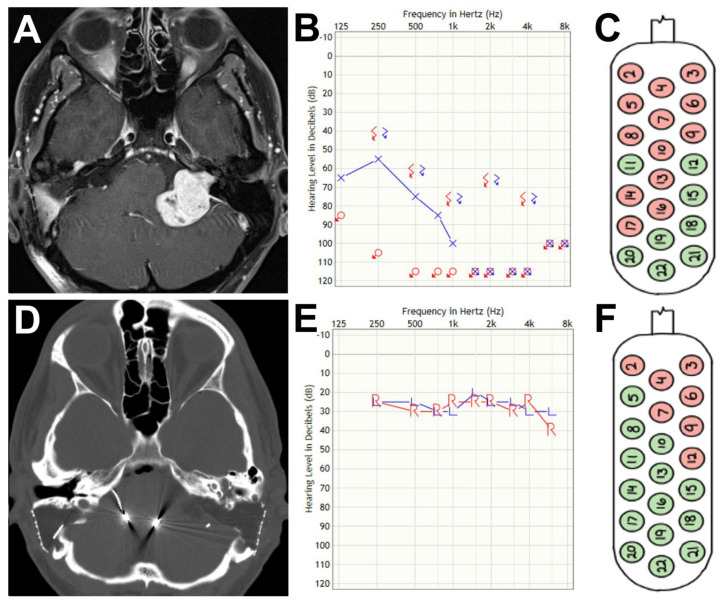
A case of bilateral ABI placement and successful audiologic outcomes. (**A**) Post-contrast T1-weighted axial magnetic resonance image through the region of the cerebellopontine angle revealing sequela of prior right sided translabyrinthine approach to tumor removal with concurrent ABI placement, now with large left cerebellopontine angle mass indicated for removal. (**B**) Pre-operative unaided audiogram prior to removal of left-sided tumor. (**C**) Right-sided electrode array map indicating 8 electrodes providing audiologic stimulation (green). (**D**) Postoperative CT brain after second-sided surgery with evidence of bilateral translabyrinthine craniotomies and bilateral ABI placement. (**E**) Postoperative audiogram revealing excellent ABI-aided pure tone thresholds for both the right and left ear. (**F**) Left-sided electrode array map indicating 14 electrodes providing audiologic stimulation (green).

The remaining case of a 34-year-old man with NF2 who underwent second-sided ABI placement at our center is noteworthy (Figure 3). At the age of 18, he underwent right-sided cyberknife radiosurgery for a growing cerebellopontine angle tumor. At the age of 22, he underwent a right-sided translabyrinthine approach for removal of this tumor and ABI placement. He used the device intermittently over the course of the next ten years, despite having residual hearing on the left side. Hearing declined on the left at the age of 34 in the presence of a large left-sided CPA schwannoma (Figure 3A), and he underwent left translabyrinthine approach for tumor removal and placement of ABI at our institution, performed by the same neurosurgeon who performed his earlier right-sided tumor removal and ABI while practicing at the patient’s previous institution. Appropriate position of bilateral ABIs was confirmed on postoperative computed tomography imaging (Figure 3D). At the time of his second-sided device activation, he was experiencing auditory stimulation from 14 electrodes on the left (Figure 3C) and 8 on the right (Figure 3F). At six-month follow-up visit, he had been following a dedicated auditory rehabilitation regimen. He described having had an improvement to a more normal voice quality from initial squealing quality at activation. ABI-aided sound-field measures were improved bilaterally (Figure 3B,E). He also experienced improved speech perception as measured by hearing in noise sentence testing in each ear that was improved in auditory + visual conditions as compared to auditory only and that was further improved in the bilateral auditory + visual condition (Table 4). The use of bilateral ABIs is not standard and is specific to uncommon situations. However, with daily use of bilateral ABIs, this patient’s auditory abilities exceed those reported in previous literature.

## 4. Discussion

For patients with loss of bilateral cochlear nerve function, ABI placement can be critical in restoring sound awareness, and in some cases, improving access to speech [14]. Around 50% of patients undergo microvascular tumor resection and ABI placement via a translabyrinthine approach [15]. However, the translabyrinthine approach was used in 93.8% (15/16) of patients in this series. Intraoperative adjustment of the electrode array, guided by electrically evoked auditory brainstem responses (eABRs), was performed in 25% (4/16) of cases. While the specific frequency of such adjustments is not extensively documented in the literature, optimizing electrode placement is critical to ensure effective stimulation of the cochlear nucleus and to enhance auditory outcomes [16,17]. The observed improved subjective and objective sound awareness with ABI parallels existing literature showing ABI improves sound recognition and speech perception, especially when combined with lip-reading [18,19]. Behr et al. found open-set sentence recognition in 46% of patients with ABI only and 59.6% of patients with ABI + lip-reading [19]. Our open-set speech perception outcomes measured by CNC whole word, CNC phonomes, and HINT sentences fall into a similar range of 8–54% understanding with audio input only and 26–88% with audio and visual inputs.

There were no ABI-specific postoperative complications observed in our cohort. The most notable postoperative morbidities included facial nerve paresis in four patients (25%) and one case (6.25%) of cerebrospinal fluid leak requiring lumbar drain followed by VP shunt placement. These complications are consistent with those reported by Colletti et al., who found that transient facial palsy is reported in 22.2% (8/36) of NF2 patients and cerebrospinal fluid leaks are reported in an average of 16.7% (6/36) of NF2 patients [20]. Our study also identified two patients with persistent non-auditory sensations that can occur with ABI use; effects can typically be managed with electrode reprogramming.

An ABI can be placed using either a translabyrinthine approach or a retrosigmoid approach and relies on eABRs to appropriately position the device. eABRs are elicited using individual ABI electrodes and can be a useful intraoperative guide to optimal positioning on the cochlear nucleus complex. Some studies have singled out postoperative eABR as a better predictor of long-term efficacy of ABI compared to intraoperative eABR, as postoperative decompressive shifts in the brainstem around the site of the implant may displace the device and electrodes from the cochlear nucleus complex, resulting in a different response [21].

The position of the ABI on the cochlear nucleus is important for creating the best treatment response in the patient. Studies on mice have concluded that the stimulation of the ventral compared to the dorsal cochlear nucleus resulted in lower modulation thresholds, and if carried over to humans would allow better differentiation between vowels and words in sentences [22]. Results from a recent study compared auditory perception to the location and orientation of the implant as seen on postoperative computerized tomography. Recipients with better speech thresholds had electrode arrays that were positioned closer to the basion (midpoint of the anterior foramen magnum) at the midline, electrodes with lower charge thresholds, and arrays that were tilted superiorly [23]. These results help clarify the optimal array position to stimulate the ventral cochlear nucleus to maximize the number of electrodes eliciting auditory stimulation as opposed to non-auditory sensations of the ABI.

Higher numbers of active electrodes identified intraoperatively have been associated with higher probabilities of obtaining meaningful postoperative eABRs and device function [24]. Other studies have found an association between higher numbers of active electrodes and increased perceptual results, including better closed-set word recognition [21,24]. Interestingly, children were found to require fewer electrodes to increase their hearing, outperforming adults in every metric, presumably due to increased neural plasticity [21]. In our analysis, we found a non-significant association between the number of active electrodes and presence of open-set speech recognition (*p* = 0.09). More complex regression analyses were precluded due to the use of multiple and different measures to assess speech perception in our dataset, which were chosen based on individual patient hearing outcomes, ranging from simple sound awareness up to open-set speech recognition. We did not find an association between number of active electrodes and current user status, though this was likely confounded by the presence of intact hearing on the contralateral side in all six of the non-users in our study. The highest performers in our cohort in terms of HINT had high numbers of electrodes: one patient with 86% audio + visual HINT score had 20 active electrodes, and another with 100% had 14 active electrodes. With more active electrodes, there is room to create specific mapping allowing for further specification on frequency-specific stimulation. This mapping specificity may enable some patients to gain more than environmental awareness with the ABI, potentially enabling more meaningful speech perception. Device use and long-term outcomes vary widely among ABI recipients. Veronese et al. found 20% (5 of 24) stopped using the ABI [21]. In the present study, 37.5% (6 of 16) of patients stopped using their ABI, though this was influenced by the fact that all 6 of the non-users retained contralateral hearing and may have relied less on their ABI for auditory input.

Currently, bilateral ABI implantation is not routine, though recent studies suggest it could offer promise and help improve auditory perception for patients. A previous study of 24 patients found bilateral ABI implantation may yield improved outcomes, particularly in patients with poor performance after a first implant [25]. These results align with our finding of an exceptional bilateral ABI user with excellent open-set speech understanding. Our chief rationale for placing ABIs in patients with good hearing contralaterally is two-fold: (1) there is a historically non-trivial non-stimulation rate (5–10%) [26], and hence no guarantee that a future contralateral implant would function if needed once the remaining hearing ear declines; (2) undergoing implantation at the same time as tumor resection avoids the risk of a second future ABI-implantation surgery that would be complicated by surgical plains obscured by scar. Our primary goal is to provide at least one functional implant for NF2 patients in whom it is expected that both ears will eventually become involved. The secondary goal is to provide the possibility of bilateral functioning implants with the potential of exceptional auditory outcomes, as highlighted herein (Table 4). It is our expectation that most patients, especially teens, will be non-users if they maintain good contralateral hearing. Data on bilateral ABI implantation remains limited and future cohort studies are needed to elucidate indications, outcomes, and long-term benefits.

This retrospective review has several significant limitations. The unique indications for auditory brainstem implantation do not allow for a suitable control group for comparison of outcomes. Furthermore, there is great heterogeneity in this patient population in terms of baseline hearing, with some patients necessarily undergoing implantation at the time of tumor resection despite having intact contralateral hearing while others are implanted after having lost bilateral hearing entirely. This limits the ability to identify patient or disease factors that may be associated with audiologic outcomes, as recipients with contralateral hearing are much less likely to spend comparable time or effort to participate in ABI-related auditory rehabilitation. The wide range in auditory outcomes also presents a complicating factor in terms of audiologic assessment postoperatively, with different batteries of speech testing applied for varying degrees of audition. The use of a variety of auditory outcome measures in this way limits the generalizability of our findings. Standardization of the breadth of testing administered may help limit this shortcoming; however, patient fatigue in participating in programming and testing would remain a significant factor. The univariate regression analysis indicating a non-significant association of active electrode number with open-set speech recognition is prone to type II error given the small sample size and was examined for exploratory purposes only. Finally, our interval of follow-up is somewhat limited by the recent establishment of the ABI program at our institution and does not account for potential maximum ABI performance over time. A standardized audiologic follow-up schedule would allow for additional analysis of plateau effects of improvement for auditory awareness. There is also a need for analysis of effective aural rehabilitation techniques specific to ABI recipients. Furthermore, the model by which patients seek care at our institution places geographic limits on audiologic follow-up, with some patients being referred to local audiology groups for continuity of their future care or seeking audiologic programming on an as-needed basis.

## 5. Conclusions

Auditory brainstem implantation at our center since 2018 resulted in sound awareness in all ABI recipients and improved lip-reading in 14 of 16 patients. Among our ABI recipients, 37% were not regularly using the device at last follow-up, likely because they were continuing to rely on their retained contralateral hearing in all 6 of these cases. While we are unable to correlate specific patient or tumor factors with more successful speech perception, the outcomes achieved in this patient cohort support the conclusion that even patients with large tumors and long-standing hearing loss have the potential for meaningful improvements in audition and communication with the aid of an ABI with a high rate of efficacy. Continued research elucidating rehabilitation protocols, bilateral implantation, and long-term outcomes is essential to optimize auditory outcomes of ABI recipients.

## Figures and Tables

**Table 1:** Patient Characteristics.

Age (years)	41 (range 14–74)
Sex (% Female)	50
Side of implant (% Left)	50
Contralateral hearing present at time of ABI (%)	50
Time from surgery to activation (months)	3.6 (range 1.5 to 8)
Indication for surgery, NF2	17
Indication for surgery, Meningitis and failed CI	1
Maximum tumor dimension (cm ± SEM)	2.9 ± 0.3
Post-op House-Brackmann 1 or 2 (%)	75 (12/16)
Number of active electrodes	12.1 (range 7–20)
Sound awareness (%)	100 (16/16)
Improved lip reading (%)	87.5 (14/16)
Persistent non-auditory sensations (%)	12.5 (2/16)
Currently user at last follow-up (%)	62.5 (10/16)
Non-users with intact contralateral hearing (%)	100 (6/6)

**Table 2:** Audiological Outcomes. * indicates rare or non-use; (SNHL = sensorineural hearing loss, ABI = auditory brainstem implant, HL = hearing loss, LF = low frequency, HF = high frequency, CI = cochlear implant, MLV = monitored live voice, A = auditory, A + V = auditory + visual, V = visual, ESP = Early Speech Perception, CNC = Consonant-Nucleus-Consonant words, HINT = Hearing in Noise Test).

Participant Number	Contralateral Ear Status	Presentation Mode	Presentation Level (dB HL)	Speech Test Materials	Years of ABI Use
1	Profound SNHL/prior failed ABI	N/A—no aided testing available, only pre-op audiogram			1
2	Severe to profound SNHL	Monitored Live Voice (MLV); A, A + V	60	CNC	6
3	Profound SNHL/prior failed ABI	MLV, recorded; A, A + V	60, 50	CNC, HINT	2
4	ABI	MLV; A, A + V	50	HINT	2
5	Mild sloping to profound SNHL	MLV; A + V	55	CNC, HINT	1
6	Profound SNHL	Aided warble tone thresholds only; not enough sound awareness for speech testing to be performed			1
7	Mild HL	MLV; A, A + V, V	60	ESP, CNC	6 *
8	Profound SNHL	MLV; A, A + V, V	60	CNC, HINT	6
9	Profound SNHL	MLV	60	ESP	6 *
10	Profound SNHL	MLV; A, A + V	60	ESP, spondees, HINT	5
11	CI	MLV; A, A + V	60	ESP, CNC, AZ Bio	6
12	Mild LF HL	MLV; A, A + V	60	ESP, spondees	5 *
13	Mild LF HL to WNL	MLV; A, A + V, V	60	CNC	5 *
14	R WNL	MLV; A, A + V, V	60	ESP, CNC, AZ Bio	5 *
15	Moderate SNHL	MLV; A, A + V	60	ESP	4
16	Mild to moderate HF HL	MLV; A, A + V, V	60	CNC	4 *

**Table 3:** Speech Perception Outcomes. (CNC = Consonant-Nucleus-Consonant words, HINT = Hearing in Noise Test).

Speech Test	Audio Only % Correct ± SEM	Audio + Visual % Correct ± SEM
Pattern perception (n = 7)	74 ± 6	85 ± 6
Spondee recognition (n = 5)	48 ± 17	82 ± 9
CNC whole word (n = 7)	8 ± 4	26 ± 7
CNC phenomes (n = 7)	20 ± 5	50 ± 7
HINT sentences (n = 4)	54 ± 15	88 ± 7

**Table 4:** Hearing in Noise Testing for Bilateral User. (ABI = auditory brainstem implant, DNT = did not test).

	Testing Condition (Monitored Live Voice)	1 Month Post-Activation	6 Months Post-Activation
Right ABI Only	Auditory Only	14%	63%
	Auditory + Visual	93%	96%
Left ABI Only	Auditory Only	40%	84%
	Auditory + Visual	92%	100%
Bilateral ABI	Auditory Only	71%	92%
	Auditory + Visual	98%	DNT

## Data Availability

The de-identified data that support the findings of this study are available from the corresponding author upon reasonable request. This study was determined to be exempt from full Institutional Review Board review; therefore, data sharing is limited to de-identified datasets to ensure participant privacy and compliance with applicable regulations.

## Abbreviations

The following abbreviations are used in this manuscript:

ABI	Auditory Brainstem Implant
CNC	Consonant-Nucleus-Consonant
eABR	Electrical-evoked Auditory Brainstem Response
FDA	Federal Drug Administration
HINT	Hearing in Noise Test
NF2	Neurofibromatosis type 2
